# Ranibizumab for macular edema secondary to retinal vein occlusion: a meta-analysis of dose effects and comparison with no anti-VEGF treatment

**DOI:** 10.1186/s12886-015-0017-z

**Published:** 2015-03-29

**Authors:** Wei-tao Song, Xiao-bo Xia

**Affiliations:** Department of Ophthalmology, Xiangya Hospital, Central South University, 87 Xiangya Road, Changsha, 410008 China

**Keywords:** Intravitreal ranibizumab, Macular edema, Retinal vein occlusion

## Abstract

**Background:**

To compare the efficacy and tolerability of intravitreal ranibizumab (IVR) 0.5 mg or 0.3 mg with non-anti-vascular endothelial growth factor (VEGF), and to compare the efficacy of IVR 0.5 mg with IVR 0.3 mg in the treatment of macular edema secondary to retinal vein occlusion.

**Methods:**

Relevant studies were selected after an extensive search using the PubMed, EMBASE, Web of Science, and Cochrane Library databases. Outcomes of interest included visual outcomes, anatomic variables, and adverse events.

**Results:**

Four randomized controlled trials (RCTs) met our inclusion criteria. IVR 0.5 mg produced a significantly higher improvement in visual acuity at six months, with pooled weighted mean differences (WMDs) of 12.30 early treatment diabetic retinopathy study (ETDRS) letters (95% CI:10.03, 14.58) (*P* < 0.001),and led to a higher proportion of patients gaining ≥15 letters (RR, 2.36; 95%CI: 1.86, 2.99; *P* < 0.001) at the follow-up endpoint, compared with non-anti-VEGF. A more obvious reduction in central foveal thickness (CFT) was observed in the IVR 0.5 mg group than the non-anti-VEGF group, and the mean difference in CFT was statistically significant (WMD, −216.86 μm; 95%CI: −279.01, −154.71; *P* < 0.001). A similar efficacy was found between the IVR 0.3 mg group and the non-anti-VEGF group. No significant differences were found between IVR 0.5 mg and 0.3 mg. The incidence of iris neovascularization in the non-anti-VEGF group was significantly higher than that of the IVR group.

**Conclusions:**

IVR 0.5 mg or 0.3 mg was more effective than sham injection and laser treatment. IVR 0.3 mg is as effective as IVR 0.5 mg in the treatment of macular edema secondary to retinal vein occlusion.

## Background

Retinal vein occlusion (RVO) is a common retinal vascular disorder in which different complications, including macular edema, may develop with a consequent loss of central vision [1]. According to the localization of venous occlusion, central retinal vein occlusion (CRVO) and branch retinal vein occlusions (BRVO) are the most frequently occurring and clinically relevant types [2]. Both could result in macular edema, a condition that is characterized by the collection of fluid within the retina, resulting from the breakdown of the blood-retinal barrier and leakage of fluid from the vasculature [3]. It has been proven that vascular endothelial growth factor (VEGF) plays a crucial role in this pathological process [3]. VEGF expression is up-regulated by hypoxia and a number of other stimuli, and was noted to be elevated in the ocular fluids of patients with CRVO [4]. Moreover, intravitreal VEGF levels were observed to correlate with the severity of clinical findings [5]. Therefore, several anti-VEGF agents, including ranibizumab, bevacizumab, and pegaptanib, have been widely used for treating macular edema [6-9].

Among all the anti-VEGF agents, ranibizumab is a high-affinity recombinant Fab, which neutralizes all isoforms of VEGF [10]. It has been reported to provide rapid and continuous improvements in best-corrected visual acuity (BCVA) and rapid reduction of retinal thickness in the treatment of macular edema [6,7,11]. Two treatment (0.5 mg or 0.3 mg) regimens are often used when administering ranibizumab. It is unknown whether the 0.3 mg and 0.5 mg groups may have had even better outcomes. It is necessary to determine which dosage is optimal.

Some studies have evaluated the efficacy of intravitreal ranibizumab (IVR) for treating macular edema in patients with RVO [6,7,12,13]. Two previous meta-analysis about treating macular edema secondary to RVO has been published. However, they both focus on all kinds of methods, including ranibizumab, bevacizumab, and intravitreal dexamethasone, for treating this condition [14,15]. Moreover, they did not discuss the effect of different doses of ranibizumab. Recently, a high-quality study on the effectiveness of ranibizumabin treating macular edema secondary to RVO was published [6]. Therefore, we performed an updated meta-analysis based only on randomized controlled trials (RCTs) to compare the efficacy of ranibizumab in conjunction with non-anti-VEGF (sham or laser), and the efficacy of ranibizumab 0.5 mg treatment with ranibizumab 0.3 mg.

## Methods

### Literature search

A literature search of the PubMed, ISI Web of Science, EMBASE, and Cochrane library databases was performed to identify relevant studies. The search combined terms related to drugs (ranibizumab, Lucentis) and terms related to diseases (macular edema, retinal vein occlusion), with a filter restricting the results to only clinical trials. Google Scholar and the websites of professional associations were also searched for information. Once relevant articles were identified, their reference lists were searched for additional articles. The final search was carried out in April 2014 without restricting the publication year, language, or methodology.

### Inclusion and exclusion criteria

We included full-text publications when the following inclusion criteria were met: (i) study design—randomized clinical trials; (ii) population—patients with macular edema secondary to RVO; (iii) intervention—IVR 0.5 mg versus no anti-VEGF treatment, or IVR 0.3 mg versus no anti-VEGF treatment, or IVR 0.5 mg versus IVR 0.3 mg treatment; (iv) outcome variables—evaluating at least one of the outcomes of interest mentioned below; (v) duration—minimum follow-up time was 6 months. Trials were excluded if (i) they were editorials, letters to the editor, review articles, case reports, meeting abstracts, or animal experimental studies; or (ii) they were extensions of the core study with different sample sizes.

### Outcome measures

The outcomes included were: (1) the mean changes in BCVA using ETDRS charts at four meters from the baseline with different inventions, indicating functional improvement (continuous); (2) the proportion of patients who gained or lost ≥ 15 ETDRS letters at the follow-up endpoint (dichotomous); (3) the mean changes in central foveal thickness (CFT) from the baseline with different inventions on ocular coherence tomography (OCT), indicating anatomical improvement; and (4) the incidence of adverse events.

### Data extraction

Two reviewers (WTS and XBX) independently selected and assessed the methodological quality of the studies and performed the data collection in a standardized way. Disagreements were resolved through discussions and the achievement of consensus. The following data were extracted from each study: first author, year of publication, study design, location of the trial, follow-up period, sample size, type of diagnosis, treatment regimen, baseline patient characteristics, inclusion and exclusion criteria, dosage, and outcome. Patients reporting adverse effects were also recorded.

### Quality assessment

The quality assessment was performed according to the risk-of-bias tool outlined in the Cochrane Handbook for Systematic Reviews of Interventions (version 5.1.0) [16]. Six key aspects that influence the quality of an RCT were assessed: sequence generation, allocation concealment, patient blinding, personnel and outcome assessors, management of incomplete outcome data, and completeness of outcome reporting, as well as other potential threats to validity. For each parameter, “yes” indicated a low risk of bias, “no” indicated a high risk of bias, and “unclear” indicated an unclear or unknown risk of bias.

### Statistical analysis

Data from this meta-analysis are presented in accordance with the Preferred Reporting Items for Systematic Reviews and Meta-Analysis [17]. The weighted mean differences (WMDs) and risk ratios (RRs) were used to compare continuous and dichotomous variables, respectively. All outcomes were reported with 95% Confidence intervals (CIs). Considering the different clinical characteristics among study groups and the variation in sample sizes, we assumed that heterogeneity was present even when no statistical significance was identified, and we decided to combine data by using a random effects model to achieve more conservative estimates [18]. The statistical heterogeneity between studies was assessed using the chi-square test, and the quantity of heterogeneity was evaluated using the I^2^ statistic. We performed the subgroup analyses when comparing IVR 0.5 mg with non-anti-VEGF treatment. (1) to separately estimate effects according to the type of RVO; (2) to separately estimate effects for small (<100) and large sample sizes (>100); (3) to separately estimate effects according to the control group (sham or laser). However, when comparing IVR 0.3 mg with non-anti-VEGF treatment, this meta-analysis included only two studies, so we could not perform the subgroup analysis. The same is true when comparing IVR 0.5 mg with IVR 0.3 mg treatment. To investigate the potential for publication bias, we constructed standard funnel plots by visually examining their asymmetry. Publication bias was evaluated using Begg’s and Egger’s tests [19,20]. *P* < 0.05 was considered statistically significant. All statistical analyses were performed using Stata (version 12; Stata Corp, College Station, Texas).

## Results

### Identification of eligible studies

**Figure 1:**
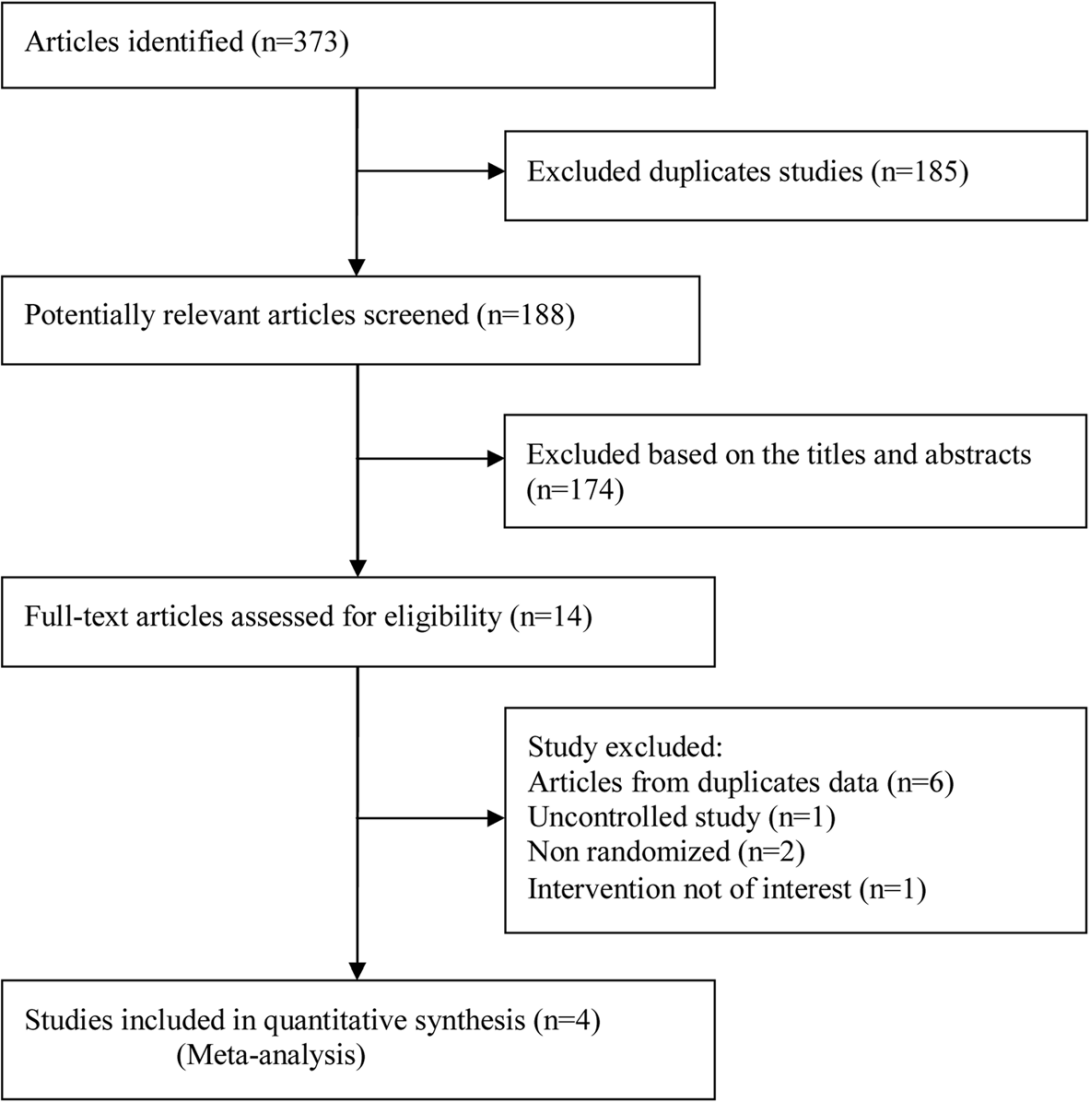
**Flowchart of publication search and selection.**

A total of 373 potentially relevant articles were identified by our literature search, of which 185 were excluded because they were duplicate studies, and 174 were excluded based on their titles and abstracts. Of the remaining 14 that were retrieved for full text review, 6 were excluded due to duplicate data [11,21-25], 2 were not randomized studies [26,27], 1 was an uncontrolled study, and the intervention of 1 was not of interest. Thus, 4 RCTs were included in the final analysis [6,7,12,13]. Interestingly, 2 articles about CRUISE and BRAVO [24,25] trials with duplicated data reported BCVA and CFT at 12 months. However, the sham control group received pro re nata (PRN) IVR 0.5 mg treatment after 6 months, which might have influenced the true effect of sham injection. So, we chose the primary endpoint for this meta-analysis [12,13]. The trial selection process is shown in Figure 1.

### Study characteristics

The main characteristics of the four included RCTs are shown in Tables 1 and 2. The trials were conducted in various countries: two were from the United States, one was from Norway, and one was from Australia. Two trials were carried out with patients with CRVO, and two were carried out with patients with BRVO. The sample size ranged from 32 to 397. Among these trials, three compared IVR 0.5 mg with sham injection, one compared IVR 0.5 mg with sham injection plus laser treatment, two compared IVR 0.5 mg with IVR 0.3 mg, and two compared IVR 0.3 mg with sham injection.

**Table 1:** **Characteristics of included RCT studies**

**Study (year)**	**Center**	**Location**	**Type of RVO**	**Primary endpoint**	**NO. of eyes**			**Age (year)**			**Sex (male/female)**			**Patient characteristics**
					**IVR 0.5 mg**	**IVR 0.3 mg**	**control**	**IVR 0.5 mg**	**IVR 0.3 mg**	**control**	**IVR 0.5 mg**	**IVR 0.3 mg**	**control**	
CRUISE (2010)	M	United States (95site)	CRVO	6 m	130	132	130	65.4 ± 13.1	69.7 ± 11.6	65.4 ± 13.1	80/50	71/61	72/58	1) Age > 18 y
														2) BCVA between 73 and 24 ETDRS letters
														3) RVO duration within 12 months
														4) CFT ≥ 250 μm
ROCC (2010)	M	Norway (4 sites)	CRVO	6 m	16		16	NA		NA	NA		NA	1) Age > 50 y
														2) BCVA between 73 and 6 ETDRS letters
														3) CFT ≥ 250 μm
BRAVO (2010)	M	United States (93site)	BRVO	6 m	131	134	132	65.2 ± 12.7	66.6 ± 11.2	65.2 ± 12.7	71/60	67/67	74/58	1) Age > 18 y
														2) BCVA between 73 to 24 ETDRS letters
														3) RVO duration within 12 months
														4) CFT ≥ 250 μm
Tan (2014)	M	Australia (5site)	BRVO	12 m	15		21	69.6 ± 11.6		66.7 ± 10.7	8/7		9/12	1) Age > 18 y
														2) BCVA between 68 to 20
														3) RVO duration between 6 weeks to 9 months.
														4) CFT ≥ 250 μm

RCT = prospective randomized controlled; RVO = retinal vein occlusion; IVR = intravitreal ranibizumab; M = multicenter; CRVO = central retinal vein occlusion; BCVA = best-corrected visual acuity; ETDRS = Early Treatment Diabetic Retinopathy Study; CFT = central foveal thickness; NA = not available; BRVO = branch retinal vein occlusion.

**Table 2:** **Characteristics of treatment exposures included in the meta-analysis**

**Trial (year)**	**Treatment group**	**Treatment protocol**
CRUISE (2010)	IVR 0.5 mg (n = 130)	IVR 0.5 mg every month for 6 months (6 injections) then PRN (open-label) for 6 months
	IVR 0.3 mg (n = 132)	IVR 0.3 mg every month for 6 months (6 injections) then PRN (open-label) for 6 months
	Sham injection (n = 130)	sham injection every month for 6 months (6 injections) then PRN IVR 0.5 mg (open-label) for 6 months
ROCC (2010)	IVR 0.5 mg (n = 16)	IVR 0.5 mg every month for 3 months, then as required (at the discretion of the physician) for persisting macular oedema
	Sham injection (n = 16)	sham injection (plastic syringe pressed against the eyeball)
BRAVO (2011)	IVR 0.5 mg (n = 131)	IVR 0.5 mg monthly injections then PRN (open-label) for 6 months
	IVR 0.3 mg (n = 134)	IVR 0.3 mg monthly injections then PRN (open-label) for 6 months
	Sham injection (n = 132)	sham injection every month for 6 months (6 injections) then PRN IVR 0.5 mg (open-label) for 6 months
Tan (2014)	IVR 0.5 mg (n = 15)	IVR 0.5 mg monthly injections up to month 5 then PRN for 6 months
	Sham injection plus laser (n = 21)	sham injection monthly up to month 5 then PRN for 6 months, laser at week 13 and 25 if eligible

IVR = intravitreal ranibizumab; PRN = Pro Re Nata.

### Quality and bias assessment of studies

The included RCTs had some risk of biases (Table 3). Sequence generation was appropriate in three studies, and allocation concealment was described in two studies. In the other two studies, these were unclear. All studies clearly elaborated upon patient blinding. All studies were judged to have a low risk of bias from selective reporting, because it was clear from the published articles that all the main pre-specified outcomes had been reported. Only one study clearly elaborated upon the analysis of intention-to-treat.

**Table 3:** **Results of Cochrane collaboration’s tool of assessing of bias**

**Trial (year)**	**Sequence generation**	**Allocation concealment**	**Blinding**			**Adequate assessment of each outcome**	**Selective reporting avoided**	**No other bias**
			**Patient**	**Personnel**	**Assessor**			
CRUISE (2010)	Yes	Unclear	Yes	Yes	Yes	Unclear	Yes	Yes
ROCC (2010)	Unclear	Yes	Yes	Yes	Unclear	Unclear	Yes	Yes
BRAVO (2011)	Yes	Unclear	Yes	Yes	Yes	Unclear	Yes	No
Tan (2014)	Yes	Yes	Yes	Yes	Yes	Yes	Yes	Yes

### Visual outcomes

In all studies, BCVA was reported to be a mean change in ETDRS letters, and it was measured by ETDRS letters from the baseline to follow-up. Pooling the results revealed that treatment with IVR 0.5 mg significantly improved BCVA, compared with non-anti-VEGF (WMD, 12.30 ETDRS letters; 95% CI: 10.03, 14.58; *P* < 0.001) at six months. A similar result was found between the IVR 0.3 mg group and non-anti-VEGF group, with a pooled WMD of 10.26 ETDRS letters (95% CI: −2.59, 2.28). However, in the mean, BCVA did not show a significant difference between the IVR 0.5 mg group and 0.3 mg group, with a pooled WMD of 1.90 ETDRS letters (95% CI: −0.35, 4.16; *P* = 0.098). When comparing the IVR 0.5 mg group and non-anti-VEGF group, we divided the studies into subgroups according to the type of RVO (CRVO and BRVO), sample size (>100 and <100), and different non-anti-VEGF treatment (sham and sham plus laser). All subgroups showed a statistically significant difference in favor of the IVR 0.5 mg group. No substantial statistical heterogeneity was observed across studies (Table 4).

**Table 4:** **Mean change from baseline in BCVA at 6 months**

**Outcome of interest**	**Studies (n)**	**WMD (95% CI)**	***P***	**Study heterogeneity**		
				χ^**2**^	***P***	**I** ^**2**^
**IVR 0.5 mg vs non-anti-VEGF**						
Type of RVO						
All trials	4	12.30 (10.03,14.58)	<0.001	1.66	0.646	0.00%
CRVO	2	14.05 (10.54, 17.56)	<0.001	0.02	0.900	0.00%
BRVO	2	11.05 (8.07, 14.02)	<0.001	0.01	0.933	0.00%
Sample size						
All trials	4	12.30 (10.03,14.58)	<0.001	1.66	0.646	0.00%
>100	2	12.43 (9.40, 15.46)	<0.001	1.61	0.205	37.9%
<100	2	11.74 (3.98, 19.51)	<0.001	0.03	0.868	0.00%
Different non-anti-VEGF treatment						
All trials	4	12.30 (10.03,14.58)	<0.001	1.66	0.646	0.00%
Sham	3	12.37 (10.02, 14.72)	<0.001	1.62	0.446	0.00%
Sham plus laser	1	11.40 (2.64, 20.16)	0.011		-	
**IVR 0.5 mg vs IVR 0.3 mg**	2	1.90 (−0.35, 4.16)	0.098	0.05	0.831	0.00%
**IVR 0.3 mg vs non-anti-VEGF**	2	10.26 (7.80, 12.72)	<0.001	1.11	0.293	9.50%

BCVA = best-corrected visual acuity; WMD = weighted mean differences; OR = odds ratio; CI = confidence interval; IVR = intravitreal ranibizumab; VEGF = vascular endothelial growth factor; RVO = retinal vein occlusion; CRVO = central retinal vein occlusion; BRVO = branch retinal vein occlusion.

**Figure 2:**
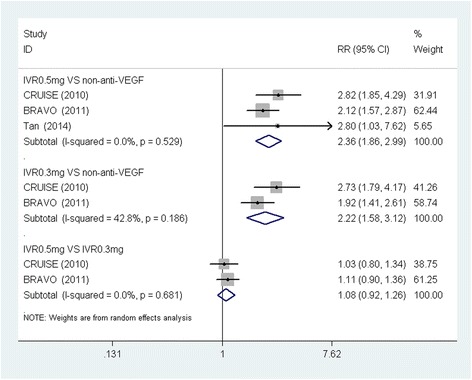
**Forest plot depicting the meta-analysis for the proportion of patients who gained ≥ 15 ETDRS letters.** RR = risk ratio; CI = confidence interval; IVR = intravitreal ranibizumab; VEGF = Vascular endothelial growth factor.

**Figure 3:**
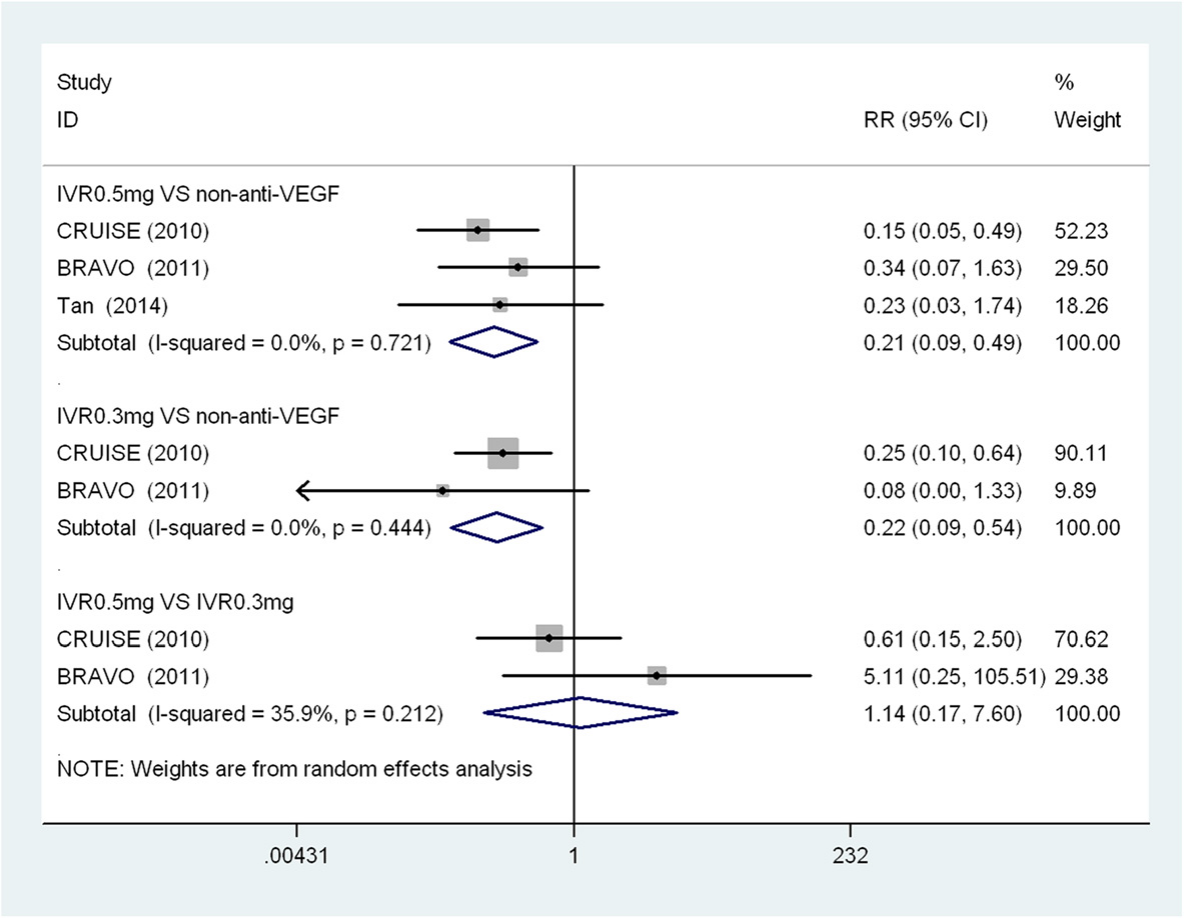
**Forest plot depicting the meta-analysis for the proportion of patients who loss ≥ 15 ETDRS letters.** RR = risk ratio; CI = confidence interval; IVR = intravitreal ranibizumab; VEGF = Vascular endothelial growth factor.

Figure 2 shows that the IVR 0.5 mg demonstrated a higher proportion of patients who gained ≥15 ETDRS letters, compared to non-anti-VEGF (RR, 2.36; 95% CI: 1.86, 2.99; *P* < 0.001) at the follow-up endpoint. IVR 0.3 mg achieved a similar result when compared with the non-anti-VEGF group (RR, 2.22; 95%CI: 1.58, 3.12; *P* < 0.001). However, no significant differences were found between IVR 0.5 mg and 0.3 mg and the proportion of patients who gained ≥15 ETDRS letters (RR, 1.08; 95% CI: 0.92, 1.26; *P* < 0.001). No substantial heterogeneity was found in these comparisons. Figure 3 shows the results of the proportion of patients who lost ≥ 15 ETDRS letters by the follow-up endpoint. Non-anti-VEGF had a higher proportion, compared to IVR 0.5 mg and 0.3 mg. No significant differences were found between IVR 0.5 mg and 0.3 mg. There was no significant heterogeneity in this analysis.

### Central foveal thickness

CFT is considered to be a strong prognostic measure for ME levels, so it was also assessed in this meta-analysis. At six months, the pooled result was more effective in decreasing CFT in the IVR 0.5 mg group, and the summary mean difference (WMD: −216.86 μm) was statistically significant (95% CI: −279.01, −154.71; *P* < 0.001), compared to the non-anti-VEGF group. A more obvious reduction in the CFT of the IVR 0.3 mg group was observed than in the CFT of the non-anti-VEGF group, and the mean difference in CFT was statistically significant (WMD, −218.97 μm; 95% CI: −303.31, −134.63; *P* < 0.001); however, the corresponding I^2^ value was 70.5%. No significant differences were found between the IVR 0.5 mg and 0.3 mg groups (WMD: −12.26 μm; 95% CI: −55.60, 31.08; *P* = 0.579). Subgroup analyses comparing the IVR 0.5 mg group to the non-anti-VEGF group were performed. All the analyses showed a statistically significant difference in favor of the IVR 0.5 mg group. The pooled estimates for the CFT change from the baseline to 6 months are summarized in Table 5.

**Table 5:** **Mean change from baseline in CFT at 6 months**

**Outcome of interest**	**Studies (n)**	**WMD (95% CI)**	***P***	**Study heterogeneity**		
				χ^**2**^	***P***	**I** ^**2**^
**IVR 0.5 mg vs non-anti-VEGF**						
Type of RVO						
All trials	4	−216.86 (−279.01, −154.71)	<0.001	5.57	0.135	46.1%
CRVO	2	−230.84 (−357.63, −104.06)	<0.001	3.03	0.082	67.0%
BRVO	2	−190.05 (−243.33, −136.78)	<0.001	0.07	0.789	0.00%
Sample size	4	−216.86 (−279.01, −154.71)	<0.001	5.57	0.135	46.1%
>100	2	−233.77 (−328.83, −138.72)	<0.001	4.49	0.034	77.7%
<100	2	−175.95 (−277.40, −74.50)	<0.001	0.30	0.585	0.00%
Different non-anti-VEGF treatment						
All trials	4	−216.86 (−279.01, −154.71)	<0.001	5.57	0.135	46.1%
Sham	3	−216.42 (−292.99, −139.85)	<0.001	5.56	0.062	64.0%
Sham plus laser	1	−210.84 (−371.90, −49.79)	<0.001		-	
**IVR 0.5 mg vs IVR 0.3 mg**	2	−12.26 (−55.60, 31.08)	0.579	0.06	0.812	0.00%
**IVR 0.3 mg vs non-anti-VEGF**	2	−218.97 (−303.31, −134.63)	<0.001	3.39	0.066	70.5%

CFT = central foveal thickness; WMD = weighted mean differences; CI = confidence interval; IVR = intravitreal ranibizumab; VEGF = vascular endothelial growth factor; RVO = retinal vein occlusion; CRVO = central retinal vein occlusion; BRVO = branch retinal vein occlusion.

### Adverse events

**Figure 4:**
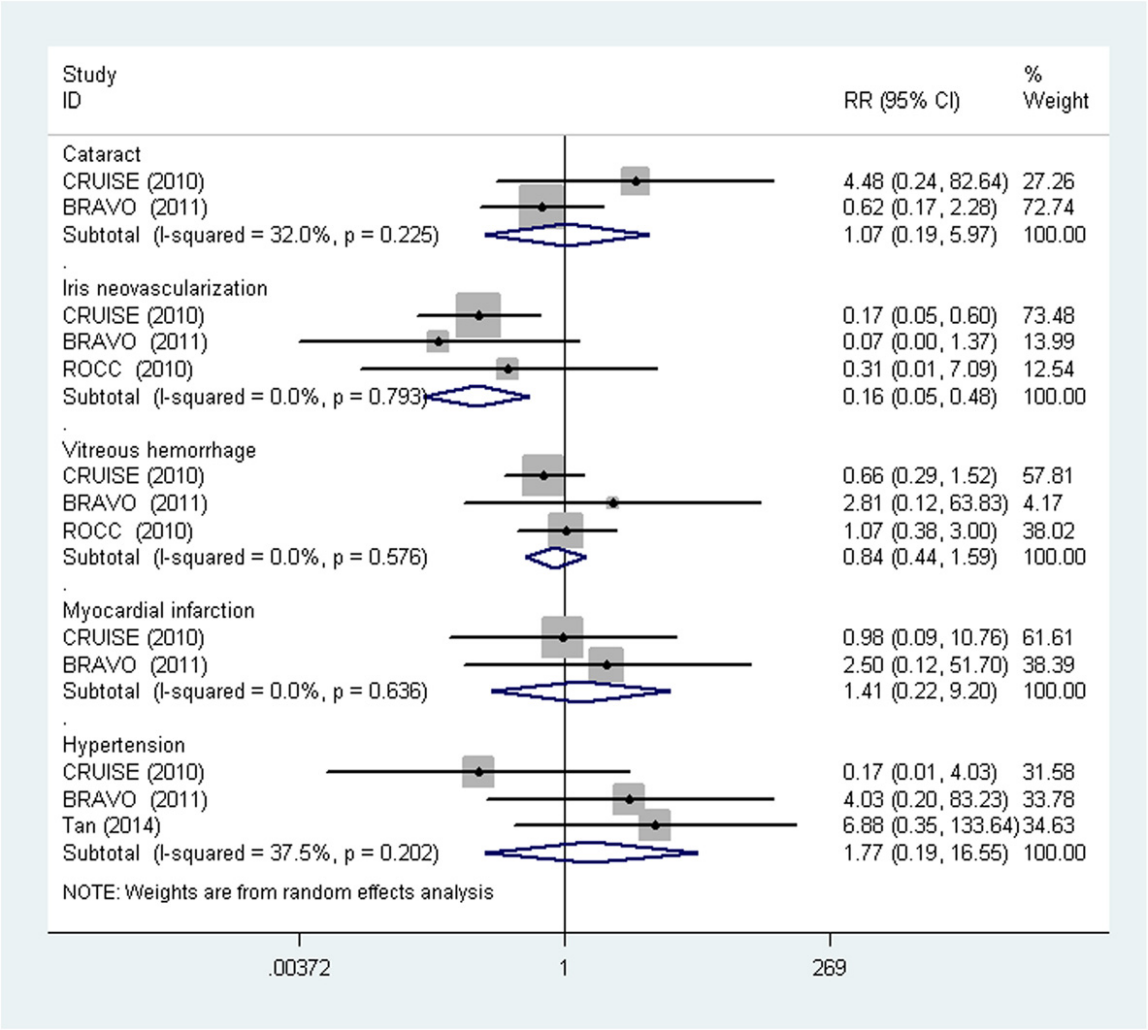
**Forest plot depicting the meta-analysis for adverse events between IVR and non-anti-VEGF treatments.** RR = risk ratio; CI = confidence interval.

We compared the incidence of adverse events, combining the IVR 0.5 mg and IVR 0.3 mg groups with the IVR group. There was insufficient data about adverse effects, restricting the ability of meta-analyses to evaluate the efficacy of adverse effects occurring at different follow-up points. The meta-analysis could only analyze the adverse events reported by two trials or more; therefore, we pooled the eye and non-ocular adverse events of cataract formation, iris neovascularization, vitreous hemorrhage, hypertension, and myocardial infarction (Figure 4). The pooled results showed that the incidence of cataract formation (RR: 1.07; 95% CI: 0.19, 5.97), vitreous hemorrhages (RR: 0.84; 95% CI: 0.44, 1.59), myocardial infarction (RR: 1.41; 95% CI: 0.22, 9.20), and hypertension (RR: 1.77; 95% CI: 0.19, 16.55) were comparable in both the IVR and non-anti-VEGF groups. However, the incidence of iris neovascularization in the non-anti-VEGF group was significantly higher than in the IVR group. No significant heterogeneity was observed in this analysis.

### Publication bias

**Figure 5:**
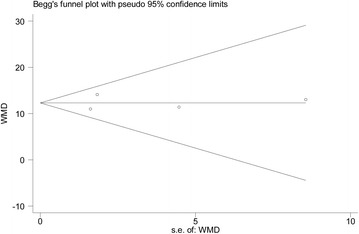
**Tests for publication bias for WMD of the BCVA change.** WMD = weighted mean differences.

A funnel plot showing the relatively symmetrical distribution suggested no evidence of publication bias, despite the small number of trials that were included in this meta-analysis (Figure 5). Begg’s and Egger’s tests, based on the mean changes of BCVA, showed that there was little potential publication bias among the included trials (Egger’s test, *P* = 0.958; Begg’s test, *P* = 0.734).

## Discussion

Ranibizumab, a high-affinity recombinant Fab, can inhibit all the biological activities of VEGF, and it has been demonstrated to be effective for treating some ocular neovascular diseases [12,13,28,29]. In the present meta-analysis, we have reviewed the literature regarding the efficacy of ranibizumab treatment for macular edema secondary to retinal vein occlusion, and regarding the success of different doses of ranibizumab for treating this visually threatening disease. Using a random-effects model, the pooled results of four RCTs indicated that ranibizumab was successful in treating macular edema secondary to retinal vein occlusion, and that 0.5 mg and 0.3 mg of ranibizumab were comparably effective.

A comparison of IVR 0.5 mg and 0.3 mg versus non-anti-VEGF showed that both IVR 0.5 mg and 0.3 mg led to marked improvements, demonstrated by the number of BCVA ETDRS letters gained and the proportion of patients who gained at least 15 letters. Three studies compared IVR with sham injection and one study compared IVR with laser coagulation treatment; both comparisons demonstrated the advantages of IVR therapy. Ranibizumab was shown to not only prevent further vision loss, but also to improve visual acuity (≥15 letters gained) among 47.7–61.1% of patients with macular edema secondary to retinal vein occlusion [6,12,13]. A similar trend was also observed in the mean CFT change; a greater reduction in foveal thickness occurred in patients treated with IVR 0.5 mg or 0.3 mg.

The pooled results of the present meta-analysis showed that the IVR 0.5 mg group was associated with a numerically larger change in BCVA and reduction in CFT, relative to the IVR 0.3 mg group. However, there was no significant difference. The results revealed that both IVR 0.5 mg and 0.3 mg treatment were effective and had the similar efficacy.

The rising popularity of ranibizumab was accompanied by concerns about its clinical safety. For example, it may result in the formation of cataracts, vitreous hemorrhages, and arterial thromboembolic events [6,12,13,25]. A comparison of the incidence of adverse events in the IVR group and non-anti-VEGF group showed that there was a higher incidence of iris neovascularization in the non-anti-VEGF group. Unsurprisingly, without the anti-VEGF treatment, persistently high concentrations of VEGF in the vitreous and anterior chambers would result in iris neovascularization. Some have raised concerns about the potential for arterial thromboembolic events with IVR treatment, but the present analysis suggests that the incidence of myocardial infarction was comparable between the two groups. Moreover, Yanagida et al. [30] undertook a systematic review of the adverse events associated with ranibizumab and found that it was systemically safe for treating macular edema. Thus, the conclusion could be drawn that IVR was a safe treatment for macular edema secondary to retinal vein occlusion. However, physicians should pay attention when administering repeated doses.

For the current meta-analysis, we evaluated the efficacy and safety of IVR 0.5 mg and 0.3 mg for treating macular edema secondary to retinal vein occlusion. In an attempt to produce robust results, we stated rigorous inclusion criteria before beginning and included only RCTs that compared the efficacy of ranibizumab and non-anti-VEGF for treating this disease. Moreover, we performed subgroup analyses, the results of which suggested that all subgroups did not materially alter the pooled results, adding robustness to our main findings. In this analysis, most comparisons exhibited no heterogeneity.

Our study had a number of strengths. First, all the original studies that were included used a randomized controlled design. Second, the present meta-analyses had strict inclusion and exclusion criteria. Third, in the CRUISE [13] and BRAVO [12] studies, the sham injection group received sham injections every month for 6 months, then PRN IVR 0.5 mg for 6 months. Thus, we only extracted the outcome of month 6 to avoid crossover of the treatment groups. Fourth, we strictly followed the Cochrane Handbook for Systematic Reviews of Interventions and the PRISMA statement when performing the literature search, data extraction, quality assessment, and statistical analysis. This makes our conclusions more scientific and reliable. Thus, this meta-analysis contributes robust information to this area of study.

Although the present analysis represents a complete summary of the currently available evidence for the efficacy of IVR 0.5 mg or 0.3 mg for treating macular edema secondary to retinal vein occlusion, it also serves to highlight any limitations. The main weakness of the analysis is the short follow-up times of the included studies. Longer follow-ups after the last injection or longer periods of repeated injections would have provided more certainty regarding treatment recommendations. Another potential limitation is that the included trials contained two types of RVO. This factor may result in heterogeneity, and could impact our results. However, subgroup analyses showed that the CRVO and BRVO subgroups had similar results and did not alter the pooled results. Additionally, the small number of trials eligible for our meta-analysis made it difficult to acquire enough data for meaningful results. Fourth, the sample sizes of some of the included studies were small. For example, Tan’s [6] trial only included 36 participants, which substantially increases the risk of a type II error. The fifth limitation is that the analyses of adverse events outcome measures were based on data pooled from trials with different follow-up periods. Finally, as we cannot attempt to gain access to unpublished results, publication bias cannot be fully excluded.

## Conclusions

In conclusion, the current limited evidence suggests that ranibizumab is more effective than sham injection and laser treatment. IVR 0.3 mg is as effective as IVR 0.5 mg for treating macular edema secondary to retinal vein occlusion. However, long-term data on the effectiveness and safety of this treatment method are needed.

## References

[1] Laouri M, Chen E, Looman M, Gallagher M (2011). The burden of disease of retinal vein occlusion: review of the literature. Eye (Lond).

[2] Wong TY, Scott IU (2010). Clinical practice Retinal-vein occlusion. N Engl J Med.

[3] Vinores SA, Derevjanik NL, Ozaki H, Okamoto N, Campochiaro PA (1999). Cellular mechanisms of blood-retinal barrier dysfunction in macular edema. Doc Ophthalmol.

[4] Aiello LP, Avery RL, Arrigg PG, Keyt BA, Jampel HD, Shah ST (1994). Vascular endothelial growth factor in ocular fluid of patients with diabetic retinopathy and other retinal disorders. N Engl J Med.

[5] Boyd SR, Zachary I, Chakravarthy U, Allen GJ, Wisdom GB, Cree IA (2002). Correlation of increased vascular endothelial growth factor with neovascularization and permeability in ischemic central vein occlusion. Arch Ophthalmol.

[6] Tan MH, McAllister IL, Gillies ME, Verma N, Banerjee G, Smithies LA (2014). Randomized controlled trial of intravitreal ranibizumab versus standard grid laser for macular edema following branch retinal vein occlusion. Am J Ophthalmol.

[7] Kinge B, Stordahl PB, Forsaa V, Fossen K, Haugstad M, Helgesen OH (2010). Efficacy of ranibizumab in patients with macular edema secondary to central retinal vein occlusion: results from the sham-controlled ROCC study. Am J Ophthalmol.

[8] Rajendram R, Fraser-Bell S, Kaines A, Michaelides M, Hamilton RD, Esposti SD (2012). A 2-year prospective randomized controlled trial of intravitreal bevacizumab or laser therapy (BOLT) in the management of diabetic macular edema: 24-month data: report 3. Arch Ophthalmol.

[9] Wroblewski JJ, Wells JR, Adamis AP, Buggage RR, Cunningham EJ, Goldbaum M (2009). Pegaptanib sodium for macular edema secondary to central retinal vein occlusion. Arch Ophthalmol.

[10] Ferrara N, Damico L, Shams N, Lowman H, Kim R (2006). Development of ranibizumab, an anti-vascular endothelial growth factor antigen binding fragment, as therapy for neovascular age-related macular degeneration. Retina.

[11] Heier JS, Campochiaro PA, Yau L, Li Z, Saroj N, Rubio RG (2012). Ranibizumab for macular edema due to retinal vein occlusions: long-term follow-up in the HORIZON trial. Ophthalmology.

[12] Campochiaro PA, Heier JS, Feiner L, Gray S, Saroj N, Rundle AC (2010). Ranibizumab for macular edema following branch retinal vein occlusion: six-month primary end point results of a phase III study. Ophthalmology.

[13] Brown DM, Campochiaro PA, Singh RP, Li Z, Gray S, Saroj N (2010). Ranibizumab for macular edema following central retinal vein occlusion: six-month primary end point results of a phase III study. Ophthalmology.

[14] Pielen A, Feltgen N, Isserstedt C, Callizo J, Junker B, Schmucker C (2013). Efficacy and safety of intravitreal therapy in macular edema due to branch and central retinal vein occlusion: a systematic review. PLoS One.

[15] Glanville J, Patterson J, McCool R, Ferreira A, Gairy K, Pearce I (2014). Efficacy and safety of widely used treatments for macular oedema secondary to retinal vein occlusion: a systematic review. BMC Ophthalmol.

[16] Higgins J, Green S. Cochrane Handbook for Systematic Reviews of Interventions. Version 5.1.0 (updated March 2011): Section 7.7.3.5 The Cochrane Collaboration 2008. Available at: http://www.cochrane-handbook.org/. Accessed May 05, 2008.

[17] Moher D, Liberati A, Tetzlaff J, Altman DG (2009). Preferred reporting items for systematic reviews and meta-analyses: the PRISMA statement. J Clin Epidemiol.

[18] DerSimonian R, Laird N (1986). Meta-analysis in clinical trials. Control Clin Trials.

[19] Egger M, Davey SG, Schneider M, Minder C (1997). Bias in meta-analysis detected by a simple, graphical test. BMJ.

[20] Begg CB, Mazumdar M (1994). Operating characteristics of a rank correlation test for publication bias. Biometrics.

[21] Thach AB, Yau L, Hoang C, Tuomi L (2014). Time to Clinically Significant Visual Acuity Gains after Ranibizumab Treatment for Retinal Vein Occlusion: BRAVO and CRUISE Trials. Ophthalmology.

[22] Campochiaro PA, Sophie R, Pearlman J, Brown DM, Boyer DS, Heier JS (2014). Long-term outcomes in patients with retinal vein occlusion treated with ranibizumab: the RETAIN study. Ophthalmology.

[23] Varma R, Bressler NM, Suner I, Lee P, Dolan CM, Ward J (2012). Improved vision-related function after ranibizumab for macular edema after retinal vein occlusion: results from the BRAVO and CRUISE trials. Ophthalmology.

[24] Campochiaro PA, Brown DM, Awh CC, Lee SY, Gray S, Saroj N (2011). Sustained benefits from ranibizumab for macular edema following central retinal vein occlusion: twelve-month outcomes of a phase III study. Ophthalmology.

[25] Brown DM, Campochiaro PA, Bhisitkul RB, Ho AC, Gray S, Saroj N (2011). Sustained benefits from ranibizumab for macular edema following branch retinal vein occlusion: 12-month outcomes of a phase III study. Ophthalmology.

[26] Pacella E, Pacella F, La Torre G, Impallara D, Malarska K, Brillante C (2012). Testing the effectiveness of intravitreal ranibizumab during 12 months of follow-up in venous occlusion treatment. Clin Ter.

[27] Figueroa MS, Contreras I (2012). Potential anti-vascular endothelial growth factor therapies for central retinal vein occlusion. Drugs.

[28] Menke MN, Zinkernagel MS, Ebneter A, Wolf S (2014). Functional and anatomical outcome of eyes with neovascular age-related macular degeneration treated with intravitreal ranibizumab following an exit strategy regimen. Br J Ophthalmol.

[29] Sawada T, Kakinoki M, Wang X, Kawamura H, Saishin Y, Ohji M (2014). Bimonthly injections of ranibizumab for age-related macular degeneration. Graefes Arch Clin Exp Ophthalmol.

[30] Yanagida Y, Ueta T (2014). Systemic safety of ranibizumab for diabetic macular edema: meta-analysis of randomized trials. Retina.

